# Prognostic factors in metastatic renal cell carcinoma: metastasectomy as independent prognostic variable

**DOI:** 10.1038/sj.bjc.6603327

**Published:** 2006-08-29

**Authors:** U M Vogl, H Zehetgruber, M Dominkus, M Hejna, C C Zielinski, A Haitel, M Schmidinger

**Affiliations:** 1 Department of Medicine I, Clinical Division of Oncology, University Hospital, Waehringer Guertel 18-20, Vienna A-1090, Austria; 2 Department of Orthopedics, University Hospital, Vienna, Austria; 3 Clinical Division of Pathology, University Hospital, Vienna, Austria

**Keywords:** metastatic renal cell carcinoma, prognosis, prediction, targeted therapies, metastasectomy

## Abstract

Prognostic and predictive factors in patients with metastatic renal cell carcinoma (MRCC) have been evaluated from untreated patients or patients on several different treatment approaches. The aim of this analysis was to define prognostic and predictive factors in patients treated uniformly with a low-dose outpatient cytokine combination. The relationship between patient-, tumour-, and treatment-related factors was analysed in 99 patients with MRCC. These features were first examined in univariate analyses, then a stepwise modelling approach based on Cox regression was used to form a multivariate model. Nuclear grade, metastasectomy – even incomplete – C-reactive protein and lactate dehydrogenase were identified as independent prognostic factors for survival. Patients assigned to three different risk groups had statistically significant survival differences (30, 22 and 6 months, respectively). A total of 43.4% had undergone metastasectomy, mostly incomplete. Risk group affiliation was correlated with response to treatment. Our findings strongly suggest the consideration of metastasectomy in the management of patients with metastatic renal cell cancer undergoing either immunotherapy or targeted treatment.

Renal cell carcinoma (RCC) accounts for about 2–3% of all malignancies (Schroeder, 1998). Due to the rareness of warning signs, 25–30% of the patients present with metastatic disease at the time of diagnosis (Linehan WM, 1993). The outcome of patients with metastatic RCC (MRCC) is poor, with a 5-year survival rate of less than 10%. In the past several years, immunotherapy consisting of recombinant interleukin-2 (rIL-2) and recombinant interferon-alpha (rIFN-alpha) has been considered standard first-line treatment for patients with MRCC. Both dosage of IL-2 (Yang *et al*, 2003; Upton *et al*, 2005) and cytokine combination *vs* cytokine monotherapy (Blay *et al*, 1992) have been the topic of several phase III investigations. Some authors pointed out the benefit of high-dose IL-2 in terms of response and quality of response (Yang *et al*, 2003; Upton *et al*, 2005), while others found the combination of both cytokines, IL-2 and INF-alpha, crucial for response achievement (Blay *et al*, 1992). However, the majority of investigators were in complete agreement in that intravenous cytokine administration may cause severe toxicity and should therefore be restricted to a subset of patients with excellent performance status. Moreover, it appeared that patient- and/or tumour-related prognostic factors might have more impact on survival than the choice of treatment.

Several patient- or tumour-related parameters have been identified as prognostic factors for survival in MRCC. Among these were nephrectomy, baseline haemoglobin, baseline lactate dehydrogenase (LDH), C-reactive protein (CRP), alkaline phosphatase (aP), location and number of metastasis, performance status, recent weight loss and neutrophil count (Neves *et al*, 1988; Ljungberg *et al*, 1995; Citterio *et al*, 1997; Hoffmann *et al*, 1999; Atzpodien *et al*, 2003; Motzer *et al*, 2004b). However, data from these analyses have frequently been collected from untreated patients. In contrast, data from 250 patients that had undergone prior treatment for MRCC have been recently analysed for prognosis of survival. In this analysis, Karnofsky performance status (KPS), high corrected calcium and low haemoglobin were found independent risk factors for survival (Motzer *et al*, 2004b). The authors identified three risk groups depending on the number of risk factors found in each patient. Patients with two or more risk factors had a statistically significant shorter median survival (5.4 months) when compared to patients with 0 or 1 risk factor (22.1 and 11.9 months, respectively) (Motzer *et al*, 2004b). The clinicopathological data in this investigation came from patients of 29 different treatment protocols for MRCC including chemotherapy and/or cytokines. Fifty per cent of the patients had received rIL-2 and/or rIFN-alpha; however, only two patients had prior rIL-2–rIFN-alpha combination treatment.

A few years ago, we established as a standard first-line treatment for patient with MRCC a well tolerable outpatient cytokine combination, consisting of 2 weeks rIFN-alpha followed by 2 weeks of rIL-2, both given subcutaneously (s.c.). The aim of this retrospective analysis is to define prognostic and predictive factors for response and survival of patients treated according to this regimen and to identify characteristics of responders and long-term survivors. We further will discuss how this stratification model may be relevant in the era of modern targeted agents.

## PATIENTS AND METHODS

### Patients

Data from 99 consecutive patients who presented with MRCC at our department entered this analysis. All patients were treated according to our standardised outpatient-cytokine protocol as described below. Patients were recruited for outpatient immunotherapy between April 2001 and May 2005. Pretreatment characteristics, first date of treatment, response, time to progression (TTP) and date of last follow-up or death were recorded for all patients. Assessment of extent of disease consisted of computed tomography scan of the chest and abdomen and/or magnetic resonance imaging.

### Methods

Treatment consisted of 4.5 MU day^−1^ r-IL-2, given s.c. on days 1–4 in weeks 3, 4, 8, 9, 13 and 14 and of 6 MU day^−1^ SC rINF-alpha on days 1, 3, 5 in weeks 1, 2, 6, 7, 11 and 12. Treatment was performed unless report of disease progression.

Parameters analysed for impact on prognosis consisted of laboratory parameters, treatment-related factors, tumour-related factors and patient-related factors:

- *Laboratory parameters were:* Haemoglobin (Hb), LDH, CRP, aP. Haemoglobin was considered normal ⩾11.5 for women and ⩾13.5 for men. As for LDH, the cutoff point for statistical analysis was categorised 1.5 times upper limit of normal (i.e. 300 U l^−1^), according to the data of Motzer *et al* (1999). Alkaline phosphatase was considered normal up to 115 U l^−1^. According to the method of Hoffmann *et al* (1999), C-reactive protein serum levels were divided into two groups, one group with levels ⩾0.8 mg dl^−1^, the other group with levels <0.8 mg dl^−1^ (a level of <0.5 mg^−1^dl^−1^ is considered normal).
- *Treatment-related factors:* Patients were evaluated for palliative nephrectomy, history of metastasectomy and for response to outpatient cytokine treatment. Response and progression were defined according to Response Evaluation Criteria in Solid Tumours (RECIST criteria) (Duffaud and Therasse, 2000).
- *Tumour-related factors were:* Histological type of RCC (i.e. clear-cell *vs* non-clear-cell type), nuclear grade, stage, time from primary tumour to metastasis, number of metastatic sites and metastatic locations.
- *Patient-related factors were:* Age, sex and KPS

Statistical analysis was performed using SPSS for windows software (RE SPSS 11.0; SPSS, Chicago, IL, USA). Descriptive statistics of relevant demographic and clinical features were compiled. Survival time was evaluated using Kaplan–Meier survival curves. Differences between groups were tested using the log-rank test. Cox's regression analysis was used for multivariate analysis. All predictors with the highest *P*-value were excluded stepwise until only significant values were present. Survival time was measured from the date of diagnosis of metastasis to death or date of the latest follow-up. The time variable for the Cox model was defined as time between the date of metastasectomy – the latest occurring variable in our analysis – and the time of death or latest follow-up. A two-tailed *P*-value equal to or less than 0.05 was considered to indicate significance in all tests. The Spearman rank correlation coefficient, the Kruskal–Wallis test and the *χ*^2^ test were used to compare the variables among the groups.

## RESULTS

### Patient's characteristics

Patient's characteristics are detailed in Table 1. A total of 99 patients (male *n*=74, female *n*=25, median age: 65, range: 34–82 years) were included in our analysis. The most common metastatic location was the lung (61.4%), followed by bone (33.7%) and liver (22.8%). Most of the patients were in good KPS (KPS 100%: 69.7%, KPS 90%: (18.2%, KPS <80%: 12.1%). All patients had undergone outpatient immunotherapy consisting of rIL-2 and rIFN-alpha. The median clinical follow-up period was 2.7 years (range 6 months–16 years). The median time from diagnosis of primary kidney tumour to diagnosis of metastasis was 3.2 months (range 0–156 months). A total of 65.7% of all patients presented with metastatic disease at the time of diagnosis of kidney cancer.

### Tumour characteristics

Eighty-five per cent of the tumours were clear-cell carcinomas and 15.2% were non-clear-cell type. A total of 38.4% of the tumours were nuclear grade 3 and the most common local stage was pT3a (44.4%).

### Laboratory parameters

Haemoglobin was found above 11.5 g dl^−1^ in 79.4% of the patients. Lactate dehydrogenase was normal in 15.2%, and CRP and aP were found normal in 23.2% and 53.6% of the patients, respectively.

### Treatment-related factors

**Figure 1:**
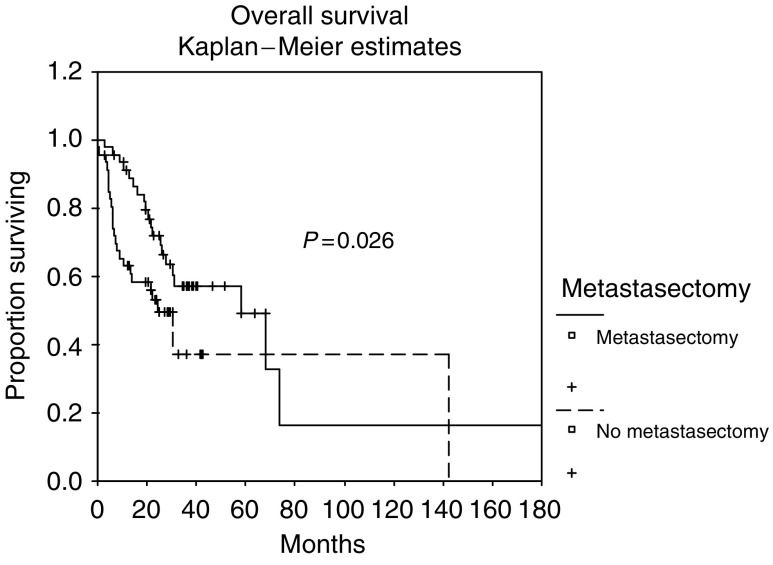
Overall survival for the independent risk factor *metastasectomy* (log rank *P*=0.026, median overall survival 27.2 *vs* 20.6 months).

A total of 92.9% had undergone nephrectomy and more than 50% had one or two metastatic sites. Objective remission to rIL-2+rIFN-alpha outpatient treatment was found in 12.8% of the patients (8.5% complete response (CR), 4.3% partial response (PR)), and another 35.2% experienced stable disease and 59.5% progressed despite treatment. Patients experiencing CR, PR or SD had a statistically significant survival benefit compared to patients progressing on treatment (median 28 and 15 months, respectively, *P*=0.0001). Figure 1 shows differences in median survival in dependence of response to treatment.

A total of 43.4% had undergone surgery for metastases (*n*=46) (27.3% bone, 11.1% lung, 4% central nervous system, 5% local recurrence, 1% pancreatic lesion). Five out of 46 patients underwent surgery for metastases twice. Table 5 outlines clinical and histological data of patients with metastasectomy compared to those without. No statistically significant differences could be observed between these two groups.

Characteristics of patients with metastasectomy and surgical techniques are detailed in Table 4. The intentions for metastasectomy were pain control and/or the management of pathological fractures (65.1%) and vital indication given by metastases to the brain in 9.3% of the patients. In 30% of the patients, the intention was to achieve a ‘no evidence of disease’ (NED) situation. However, only 21% of the patients achieved NED surgically. Causes for not achieving NED surgically (9%) were metastases to other sites diagnosed only a few days after metastasectomy (*n*=3) and unexpected incomplete resection of metastases (*n*=2). Thus, among 46 patients undergoing metastasectomy, 80% had tumour burden left after surgery and 90.7% presented with a KPS >80. Metastasectomy patients were characterised in 55.8% as high-risk patients, 37.2% presented with medium risk and 7% were in the low-risk group. Metastasectomy was performed median 6 months after diagnosis of primary tumour (range: 1–267 months). The initiation of immunotherapy was median 2 months after metastasectomy (range: 28 months prior to metastasectomy to 54 months after metastasectomy).

### Survival

The median overall survival was 22 months (range 1–280 months). The following pretreatment factors were identified as univariate predictors of poor survival:

(1) Hb level <11.5 g dl^−1^, (2) LDH level >300 U l^−1^, (3) CRP ⩾0.8 mg dl^−1^, (4) high nuclear grade, (5) brain metastasis, (6) absence of nephrectomy and (7) KPS⩽80.

In contrast, no statistically significant differences were found for the following parameters: time from diagnosis of primary tumour to metastatic disease, number of metastatic sites, other metastatic locations than brain, aP level, sex, age, stage and histological type of RCC.

Four factors were found to be significant in the multivariate analysis. As shown in Table 2, the major prognostic factor is nuclear grade (*P*=0.003, hazard ratio 3.923), followed by metastasectomy (even incomplete) (*P*=0.01, hazard ratio 0.297; Figure 1), CRP (*P*=0.034, hazard ratio 2.721) and serum LDH (*P*=0.035, hazard ratio 3.037). According to the number of multivariate risk factors, we established three risk groups and each patient was assigned to a specific risk group: low risk (zero risk factors), medium risk (one or two risk factor) and high risk (three or more risk factors).

**Figure 2:**
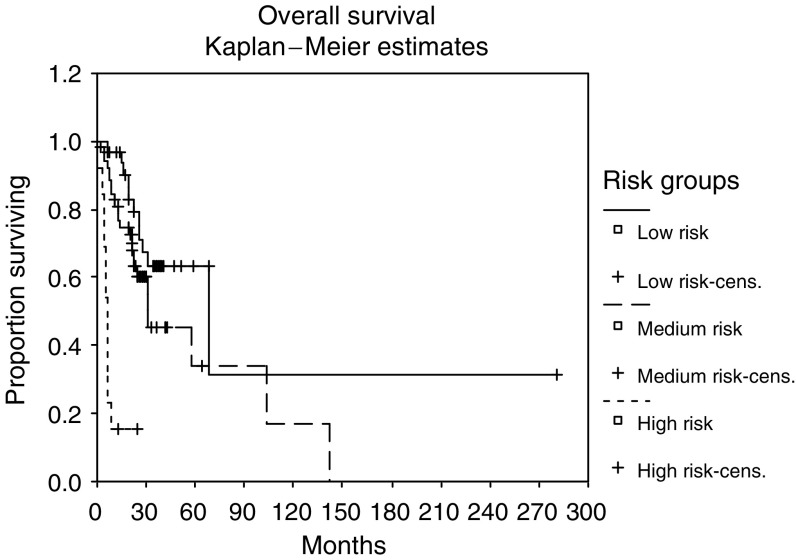
Overall survival stratified according to risk group (log rank *P*<0.001 for low *vs* medium risk and *P*<0.001 for medium *vs* high risk, median overall survival: 30.53 *vs* 22.1 *vs* 5.9 months).

There was a statistically significant difference in survival between patients in the low- (*P*<0.001), medium- and high-risk groups: 30.53 months (range: 6.4–280), 22.1 months (range: 0.43–67) and 5.9 months (range: 1–39 months), respectively (Figure 2).

### Response to immunotherapy

When comparing the three risk groups for response to treatment, we found statistically significant differences: among the patients responding to treatment, 66.7% were low, 33.3% medium risk, but no patient in the high-risk group responded to treatment (Table 3). In contrast, 57.1% of the patients who progressed were high risk, 24.5% medium risk and 18.4% low risk (*P*=0.001).

In addition, baseline Hb levels <11.5 mg dl^−1^ (*P*=0.021) and histology of non-clear-cell carcinoma (*P*=0.024) were unfavourable predictors for response. All patients with objective remissions and 97% of the patients achieving SD had baseline Hb levels of greater than 11.5 mg dl^−1^.

### TTP

Median TTP and median progression-free survival (PFS) were 6.55 months, respectively. Two factors were independent factors for time to tumour progression: high KPS and Hb levels greater than 11.5 mg dl^−1^. Patients with Hb levels lower than 11.5 mg dl^−1^ had a statistically significant shorter TTP (8.29 months) than patients with Hb levels higher than 11.5 mg dl^−1^ (3.22 months) (*P*=0.0013).

## DISCUSSION

The aim of this analysis was to define prognostic factors for response to treatment and survival in patients treated with an outpatient-cytokine regimen for MRCC. We identified nuclear grade, surgery for metastases, CRP and LDH as independent risk factors for survival. Moreover, we found statistically significant survival differences between three established (Motzer *et al*, 2004a, 2004b) risk groups (30.5, 22 and 5.9 months, respectively, *P*=0.001).

Remarkably, metastasectomy was an independent prognostic factor for survival. The benefit of surgery has frequently been demonstrated in the past, particularly in cases of singular lung metastasis with achievement of a ‘NED’ situation (van der Poel *et al*, 1999; Hofmann *et al*, 2005). However, in our analysis, only 10% of the patients who had undergone metastasectomy had NED after resection. One could argue that survival differences between metastasectomy and nonmetastasectomy patients are the result of patient selection, considering only patients with excellent performance status for this procedure. However, the intention for metastasectomy was rarely based on oncological consideration, that is, reduction of tumour burden, but was rather driven by a suddenly occurring necessity, that is patients presenting with pathological fractures from bone metastases. As shown in Table 5, the most common indication for metastasectomy was maintenance of the musculoskeletal system integrity or higher pain control (65.1%). Another indication (9.3%) was the resection of metastases to the brain otherwise causing increased intracranial pressure. Only in 30% the intention for metastasectomy was to achieve NED, which finally has been achieved in 21% only. As shown in Tables 4 and 5, the majority of our patients were in good KPS (>80%) and patients with lower KPS than 80% were found in both the metastasectomy group and the nonmetastasectomy group. Thus, we postulate that metastasectomy – even if NED is not achieved surgically – is a powerful prognostic factor in MRCC. In an attempt to explain these finding, a series of factors were considered:

High CRP is caused by excessive IL-6 production, a multifunctional cytokine with growth factor function in RCC (18). IL-6 was shown to correlate with stage, nuclear grade and proliferation index (Costes *et al*, 1997). By investigating which patients mostly benefit from palliative nephrectomy, Fujikawa and co-workers found that particularly those presenting with elevated pretreatment serum CRP levels had a benefit from nephrectomy (Matsui *et al*, 2001). Although these findings concern the removal of the primary tumour, one can hypothesise that debulking of both the primary tumour or metastatic lesions may – after a short postoperative increase – finally lower the serum level of acute phase reactants, thus lowering disease progression.

Numerous stratification models for MRCC survival have been defined in the past, with KPS, CRP, nephrectomy, Hb and nuclear grade being the most frequently identified risk factors. To date, several investigators favour the risk stratification model proposed by Motzer *et al* (2004b) with corrected calcium, haemoglobin and PFS as independent prognostic factors for survival. In this model, three risk groups with statistically significant survival differences have been described (22, 11.9 and 5.4 months, respectively). An interesting finding is that low-risk patients in our analysis had a considerable better median survival than patients of Motzer's low-risk group (30.5 *vs* 22 months, respectively). Motzer *et al* (2004b) had observed that compared to patients treated after 1990, patients treated earlier were more often categorised as high risk. We believe that survival differences between patients treated in different decades may be explained by both an improvement in surgical techniques for metastases and the advent of supportive care measures, for example, the erythropoietins.

In contrast to numerous prognostic factors, only two predictive factors for response to treatment have been identified yet: the proliferation status in terms of Ki-S5-immunoreactive scores (Papadopoulos *et al*, 1996) and the histological type of RCC (Upton *et al*, 2005). IL-2 responsiveness was shown to be predicted by clear-cell type, presence of more than 50% alveolar features and absence of papillary or granular features. In accordance with these findings, we found clear-cell histology as predictive for response to our treatment. Moreover, we could identify Hb as an independent predictor of response, which might be associated with high levels of circulating vascular endothelial growth factor (VEGF) in anaemic MRCC patients. In MRCC, erythropoietins were shown to be important for both supportive care and immunomodulatory effects: high levels of VEGF have been found in anaemic patients progressing on IL-2 treatment (Lissoni *et al*, 2001). Retreatment of the very same patients with IL-2 and erythropoietin not only lowered VEGF levels but also restored responsiveness to IL-2 in terms of achieving at least stable disease (Lissoni *et al*, 2001). Another predictive marker for response that could be identified in our analysis was the risk group affiliation. We found more responders in the low-risk group (66.7%) than in the medium-risk group and no responder in the high-risk group. Accordingly, we characterise a responder by low nuclear grade, low CRP, metastasectomy and low LDH. Particularly, CRP (and IL-6) levels have often been found to impair IL-2 efficacy (Blay *et al*, 1992; Simpson *et al*, 1995; Tartour *et al*, 1996; Thiounn *et al*, 1997). The mechanism by which these acute phase reactants interfere with IL-2-induced tumour regression is not defined. Some authors hypothesised that soluble CRP prevents recognition and binding of tumour cells by IL-2-activated effector cells (Kedar *et al*, 2004).

The question arises whether our prognostic and predictive model, established from patients undergoing cytokine treatment, may be relevant in the era of targeted therapies (Ratain, 2004; Yang, 2004; Hainsworth *et al*, 2005; Motzer *et al*, 2006). Although these agents have shown promising results in MRCC, it is questionable whether they will completely replace established strategies. It appears that their full potential may rather be achieved by combination with other agents (i.e. other targeted therapies, immunotherapy) or by combination with surgery, that is, metastasectomy. Moreover, our predictive model may offer uncommon therapeutical considerations. Prediction of response to treatment by CRP may not be restricted to immunotherapy: acute phase reactants were shown to raise plasma levels of alpha 1 acid glycoprotein, a protein that has been associated with resistance to anti-epidermal growth factor receptor (EGFR) agents (Viloria-Petit *et al*, 2001). MRCC was shown to be resistant to anti-EGFR treatment (Motzer *et al*, 2003). We hypothesise that a therapeutical reduction of CRP levels either by metastasectomy or glucocorticoids – which have been shown to inhibit VEGF-mRNA – may circumvent acute phase reactants-induced drug resistance. This phenomenon might be of particular interest when using agents that inhibit both the VEGF and EGFR signalling cascade (i.e. Sorafenib). Thus, a multimodal treatment approach that considers CRP reduction may possibly increase the efficacy of new agents.

In summary, the information gained through our prognostic and predictive model appear particularly relevant for both patients undergoing immunotherapy and patients intended for new targeted agents. It may allow an individualised treatment and the circumvention of drug resistance. However, independently from the systemical treatment approach, our data strongly suggest that even incomplete reduction of tumour burden may confer a survival benefit in MRCC. Thus, we highly emphasise a strong cooperation with surgeons in this disease. As the majority of patients (with or without metastasectomy) in this analysis had a favourable KPS, a careful patient selection for any surgical procedure seems warranted at this stage of the disease.

## Figures and Tables

**Table 1:** Clinicopathological data of 99 patients with MRCC

**Characteristics**	***n***	**Median**	**%**	**Range**
No. of patients	99			

*Sex*				
Male	74		74.7	
Female	25		25.3	
				
Age (years)		65		34–83
				
*KPS*				
100%	69		69.7	
90%	18		18.2	
⩽80%	12		12.1	
				
*Histology*				
Clear cell	85		85.9	
Papillary	12		12.1	
Collecting duct	2		2	
				
*T stage*				
pT1	15		15.2	
pT2	12		12.1	
pT3a	44		44.4	
pT3b	28		28.3	
				
*Nuclear grade*				
G1	3		3	
G2	30		30.3	
G3	38		38.4	
G4	18		18.2	
Not available	10		10.1	
				
*Nephrectomy*				
Yes	92		92.9	
No	7		7.1	
				
*No. of metastatic sites*				
Local recurrence only	3		3	
1 site	33		33.3	
2 sites	28		28.3	
3 sites	17		17.2	
⩾4 sites	18		18.2	
				
*Sites of metastatic disease*				
Lung	62		61.4	
Mediastinum	19		18.8	
Bone	34		33.7	
Liver	23		22.8	
Abdominal lymph nodes	14		18.9	
Pancreas	2		2	
Cerebral	11		10.9	
				
*Surgery for metastases*				
No metastasectomy	56		56.6	
Metastasectomy	43		43.4	
Pulmonary	11		11.1	
Bone	27		27.3	
Local recurrence surgery	5		5	
CNS	4		4	
Pancreas	1		1	
Patients receiving metastasectomy twice	5		5	
				
*Baseline laboratory parameters*				
Haemoglobin normal ⩾11.5 g/dl	78	12.5	78.8	8.8–16.3
LDH normal <200 U/l	68	176	68.7	26–2350
CRP normal <0.5 mg/dl	23	2.1	23.2	0.5–195
aP normal 0–15 U/l	53	111	53.5	47–819
				
*Best response to outpatient immunotherapy*				
CR	8		8.5	
PR	4		4.3	
SD	33		35.2	
PD	49		49.5	
Not evaluable	5		5.1	

aP=alkaline phosphatase; CNS=central nervous system; CR=complete response; CRP=C-reactive protein; KPS=Karnofsky performance status; LDH=lactate dehydrogenase; MRCC=metastatic renal cell carcinoma; PD=progressive disease; PR=partial response; SD=stable disease.

**Table 2:** Multivariate survival analysis of pretreatment clinical factors

**Risk factors**	**Categories compared**	***P*-value**	**Risk ratio**	**95% CI**
Grade	G1/G2 *vs* G3/G4	0.003	3.923	1.608–9.573
CRP	<0.8 *vs* ⩾0.8 mg dl^−1^	0.034	2.721	1.080–6.858
Metastasectomy	Yes *vs* no	0.010	0.297	0.118–0.749
LDH	<300 *vs* >300 U l^−1^	0.035	3.037	1.080–8.540

CI=confidence interval; CRP=C-reactive protein; LDH=lactate dehydrogenase.

**Table 3:** Response to immunotherapy stratified according to risk group (*P*=0.001)

	**CR, PR**		**SD**		**PD**	
	***n***	**%**	***n***	**%**	***n***	**%**
Low risk, *n*=32	8	66.7	15	46.9	9	18.4
Medium, risk *n*=32	4	33.3	16	50.0	12	24.5
High risk, *n*=29	0	0	1	3.1	28	57.1

CR=complete relapse; PD=progressive disease; PR=partial relapse; SD=stable disease.

**Table 4:** Characteristics of patients with metastasectomy

**Patient**	**Metastasectomy (location and surgical techniques)**	**Intention of metastasectomy**	**Tumour burden left after metastasectomy (other sites)**	**Time to tumour progression after complete metastasectomy**	**KPS**	**Risk group**	**Time between diagnosis and metastasectomy (months)**	**Time between metastasectomy and immunotherapy (months)**
1	Bone (hip: resection and reconstruction)	Fracture, pain control	Bone		90	Medium	60.79	1.55
2	Bone (curretage and vertebroplasty)	Fracture, pain control	Bone		90	Medium	15.63	2.01
3	Bone (humerus: curretage and plate osteosynthesis)	Fracture, pain control	Liver, lung, mediastinal lymph Nodes		100	High	0.59	2.6
4	Bone (2 curretages and vertebroplasties)	Pain control	Liver		100	Medium	1.61	0.86
5	Bone (humerus: curretage and plate osteosynthesis)	Fracture, pain control	Liver, lung		100	Medium	13.2	−11.2
6	Bone (curretage and vertebroplasty)	Pain control	Liver		100	Medium	98.52	1.84
7	Lung (segment resection), bone (hip: resection and reconstruction)	Tumour reduction	No	4.3	90	Low	0	0.56
8	Lung (segment resection)	Tumour reduction	No	9.4	100	High	267.40	0.92
9	Pancreas (whipple operation)	Tumour reduction	No	46	100	Medium	0	46.25
10	Bone (curretage and vertebroplasty)	Pain control	Lung		100	Medium	16.41	3.05
11	Bone (humerus: curretage and plate osteosynthesis)	Fracture, pain control	Bone		100	High	65.53	1.15
12	Bone (femur: curretage and osteosynthesis)	Fracture, pain control	Lung		90	High	0	2.1
13	Lung (segment resection)	Tumour reduction/ others^a^	Abdominal lymph nodes		100	High	0	1.74
14	Bone (femur, acetabulum: curretage and osteosynthesis)	Fracture, pain control	Lung		90	High	51.09	3.18
15	CNS (excision), lung (segment resection)	Vital indication	No	2	100	Medium	33.78	2.47
16	Local recurrence (tumour debulking)	Pain control	No	6	100	High	93.16	12.01
17	Bone (curretage and vertebroplasty)	Fracture, pain control	Bone		100	Medium	12.93	−13.08
18	Bone (hip: excision and reconstruction)	Fracture, pain control	Adrenal		80	High	0	2.3
19	CNS (excision)	Vital indication	Lung, bone		100	High	35.13	14.97
20	Local recurrence (tumour debulking)	Pain control	Regional tumour mass		100	Medium	48.06	−28.32
21	Bone (femur: curretage and osteosynthesis)	Fracture, pain control	Bone		100	Low	0	2.92
22	Lung (excision of pleural metastasis)	Tumour reduction	No	50.2	100	Medium	96.12	53.91
23	Bone (hip: excision and reconstruction)	Fracture, pain control	Lung		80	High	0.95	−0.33
24	CNS (excision)	Tumour reduction	Lung		100	High	110.13	−4.38
25	Bone (hip: excision and reconstruction)	Fracture, pain control	Lung		100	High	0.49	0.72
26	Lung (segment resection)	Tumour reduction	No	9.2	100	High	6.05	−5.66
27	Local recurrence (tumour debulking)	Pain control	Lung		100	Low	121.12	23.52
28	CNS (excision)	Vital indication	Lung		100	High	46.05	−22.83
29	Bone (femur: curretage and osteosynthesis)	Fracture, pain control	Bone		100	High	−5.23	6.64
30	Bone (femur: curretage and osteosynthesis)	Fracture, pain control	Lung		80	Medium	−7.04	18.75
31	Lung (segment resection)	Tumour reduction/ others^b^	Lung		100	High	90.16	−14.97
32	Bone (curretage and vertebroplasty)	Pain control	Bone		90	High	60.10	24.21
33	Bone (hip: excision and reconstruction)	Pain control	Lung		100	High	127.80	4.38
34	Bone (hip: excision and reconstruction)	Pain control	Lung		90	High	73.09	−7.27
35	Lung (segment resection)	Tumour reduction/ others^a^	Liver		90	High	0	2.37
36	Bone (hip: excision and reconstruction)	Fracture, pain control	Bone		90	High	0	2.56
37	Local recurrence	Pain control	Bone		100	High	132.24	1.61
38	Lung (segment resection)	Tumour reduction/others^a^	Bone, lung		90	Medium	−0.89	1.35
39	Bone (humerus: curretage and plate osteosynthesis)	Fracture, pain control	Liver		100	Medium	−4.38	6.35
40	Bone (femur: curretage and osteosynthesis)	Fracture, pain control	Lung		100	Medium	−0.43	1.88
41	Bone (curretage and vertebroplasty)	Pain control	Liver		80	Medium	33.95	19.11
42	Bone (hip: excision and reconstruction), lung (segment resection)	Tumour reduction, pain control	No	7	100	High	120.86	7.53
43	Local recurrence (tumour debulking), lung (lobectomy)	Tumour reduction	No	14.5	100	High	102.01	−5.8
								
Total	Bone: 27.3% Lung: 11.1% CNS: 4% Local recurrence: 5% Pancreatic lesion: 1% Metastasectomy twice: 5% Total: 43.3%	Pain control/fracture: 65.1% Vital indication: 9.3% Tumour reduction: 27%	Other sites left: 79% NED: 21%	Median: 9.2 Months range: 2–50.2	>80%: 90.7% ⩽80%: 9.3%	Low risk: 7% Medium risk: 37.2% High risk: 55.8%	Median time: 6 months after diagnosis Range: 1–267 months	Median time: 2 months after metastasectomy Range: 28 months prior – 54 months after metastasectomy

CNS=central nervous system; KPS=Karnofsky performance status.

a Metastases to other sites were diagnosed only a few days after metastasectomy.

b Unexpected incomplete resection of the metastases.

**Table 5:** Comparison of characteristics of patients with metastasectomy and patients without metastasectomy

**Characteristics**	**Metastasectomy (*n*=43)**	**No metastasectomy (*n*=56)**	***χ*^2^ test**
*KPS*			
100%	29 (67.4%)	40 (71.4%)	*P*=0.357
90%	10 (23.3%)	8 (14.3%)	
⩽80%	4 (9.3%)	8 (14.3%)	
			
Haemoglobin normal ⩾11.5 g dl^−1^	35 (81.4%)	43 (76.8%)	*P*=0.519
LDH cutoff <300 U l^−1^	37 (86%)	44 (78.6%)	*P*=0.685
CRP <8 mg dl^−1^	18 (41.9%)	23 (41.1%)	*P*=0.471
			
*Risk groups*			
Low risk	3 (7%)	29 (51.8%)	
Medium risk	16 (37.2%)	16 (28.6%)	
High risk	24 (55.8%)	5 (8.9%)	
			
*Histology*			
Clear cell	40 (93%)	45 (80.3%)	
Papillary	3 (7%)	9 (16.1%)	
Collecting duct	0	2 (3.6%)	*P*=0.173

CRP=C-reactive protein; KPS=Karnofsky performance status.
